# Androgen receptor function and targeted therapeutics across breast cancer subtypes

**DOI:** 10.1186/s13058-022-01574-4

**Published:** 2022-11-14

**Authors:** Emily A. Kolyvas, Carlos Caldas, Kathleen Kelly, Saif S. Ahmad

**Affiliations:** 1 Cancer Research UK Cambridge Institute, Department of Oncology, Li Ka Shing Centre, University of Cambridge, Cambridge, CB2 0RE UK; 2 Laboratory of Genitourinary Cancer Pathogenesis, Center for Cancer Research, National Cancer Institute, Bethesda, MD USA; 3 NIH-Oxford-Cambridge Scholars Program, Cambridge Institute for Medical Research and Department of Medicine, University of Cambridge, Cambridge, UK; 4 Breast Cancer Programme, CRUK Cambridge Centre, Cambridge, CB2 0RE UK; 5 Cambridge Breast Cancer Research Unit, NIHR Cambridge Biomedical Research Centre and Cambridge Experimental Cancer Medicine Centre, Cambridge University Hospitals NHS Foundation Trust, Cambridge, UK; 6 Department of Oncology, School of Clinical Medicine, University of Cambridge, Cambridge, CB2 0SP UK

**Keywords:** Androgen receptor, Breast cancer, Radiotherapy, DNA damage repair, Androgen deprivation therapy

## Abstract

Despite significant progress in breast cancer (BC) therapy, it is globally the most commonly diagnosed cancer and leads to the death of over 650,000 women annually. Androgen receptor (AR) is emerging as a potential new therapeutic target in BC. While the role of AR is well established in prostate cancer (PCa), its function in BC remains incompletely understood. Emerging data show that AR’s role in BC is dependent on several factors including, but not limited to, disease subtype, tumour microenvironment, and levels of circulating oestrogens and androgens. While targeting AR in PCa is becoming increasingly effective, these advances have yet to make any significant impact on the care of BC patients. However, this approach is increasingly being evaluated in BC and it is clear that improvements in our understanding of AR’s role in BC will increase the likelihood of success for AR-targeted therapies. This review summarizes our current understanding of the function of AR across BC subtypes. We highlight limitations in our current knowledge and demonstrate the importance of categorizing BC subtypes effectively, in relation to determining AR activity. Further, we describe the current state of the art regarding AR-targeted approaches for BC as monotherapy or in combination with radiotherapy.

## Background

Breast cancer (BC) is the most common cancer in women with over 2.2 million estimated new cases and over 650,000 deaths each year worldwide [1]. While substantial improvements have been made over the years to reduce BC mortality, it remains a significant cause of death in women. BC is a heterogeneous disease with many subtypes with different molecular features. These subtypes are characterized by established biomarkers such as oestrogen receptor alpha (ER), progesterone receptor (PgR), and human epidermal growth factor receptor 2 (HER2) [2]. Molecular classification is important not only for patient prognosis but also to help predict therapy response and guide treatment strategies. To improve BC treatment, there remains a need to identify novel and alternative therapeutic targets for this disease, particularly in triple-negative breast cancer (TNBC) where systemic cytotoxic chemotherapy remains the primary pharmacological intervention. Although recent evidence has established a role for immunotherapy in metastatic TNBC, these benefits appear to be limited to patients with positive PD-L1 status [3]. In this respect, the androgen receptor (AR) is emerging as a new biomarker and a potential therapeutic target in BC.

AR is shown to be involved in all stages of BC development and is expressed in up to 30–80% of BC by immunohistochemistry (IHC), depending on subtype [4]. Notably, there is a wide range of reported AR+ samples in part due to a lack of standardization of cut-off levels to determine AR positivity. The exact mechanism of AR action in BC, however, remains elusive. The role of AR likely depends on the subtype of BC as well as levels of circulating hormones and tumour microenvironment. While data suggest that AR could be used as a potential biomarker, the degree of prognostic value remains unclear, making it difficult to identify which patients would benefit from therapeutics that exploit AR as a target. Furthermore, recent data suggest that AR is associated with radiation therapy (RT) resistance. Understanding the complex role of AR in BC subtypes would be useful in predicting which patients would benefit from targeted AR therapies.

## AR biology

AR is a steroid hormone receptor that acts as a ligand-dependent transcription factor. Once the ligand binds, AR translocates to the nucleus of the cell where dimerized receptors bind to enhancer and promoter regions termed androgen response elements (AREs) of target genes. This leads to the initiation of transcription, cell proliferation and survival, and negative feedback to inactivate AR transcription [5]. Ligands that bind to AR principally include testosterone (T) and 5a-dihydrotestosterone (DHT). Other androgens such as androstenedione, androstenediol, and dehydroepiandrosterone (DHEA) have also been shown to bind AR, albeit with much less potency than T and DHT [6]. The levels of circulating androgens differ significantly between males and females. T and DHT are present at the highest concentrations in males while in females they are at the lowest compared to other androgens [7]. Given the differences in potency, the majority of AR activation in females will be likely due to T and DHT. AR also has the potential to be activated through ligand-independent mechanisms, including through interactions with PI3K/AKT, ERK, mTOR, and Wnt/β-catenin signalling pathways (reviewed in Anestis et al. [8]). AR activity is well established as a dependency of prostate cancer (PCa) throughout all stages of growth and progression, leading to the essential role of AR-directed therapies for PCa [9].

AR is expressed in various tissues in females as well, including breast tissue, and it is known to play a significant role in normal female biology, fertility, and breast development [10]. AR is also commonly expressed in BC, and while sex steroid signalling is very well established as being critical to the development of BC at all stages, the role of AR signalling remains unclear [11]. AR is broadly expressed across multiple types of BC, leading to its emergence as a target for BC therapeutics and the ongoing research exploring AR as a predictive and prognostic biomarker [12]. AR is expressed in 30–80% of BC, with more common co-expression with ER+ (70–90%) over ER– cancer (20–30%) [13–15]. The variation in expression across studies is due, in part, to differing definitions of AR positivity (Table 1). Recently, Ricciardelli et al. summarized the use of AR for BC prognosis and concluded that a higher median cut-off of AR positivity (≥ 78%) could more reliably predict BC survival compared to other commonly used cut-offs (1% or 10% nuclear positivity) [16]. Table 2 shows this variability in the definition of AR positivity across clinical trials, with a range of positive IHC staining from > 0% to 50%. The clinical significance of AR expression seems to differ based on the type of BC, which becomes more evident as we assess the prognostic value of AR by subtypes of BC.

**Table 1:** Defining AR positivity across breast cancer samples

References	ER status	HER2 status	Primary/metastatic	Antibody	AR+ definition	AR+ samples (%)	Total
Agrawal et al. [62]	ER+/ER−	±	Primary Only	AR411 DAKO	Not Reported	212 (43)	488
Gonzalez et al. [63]	ER+/ER−	Not reported	Primary & Lymph Node Metastases	AR411 DAKO	≥ 1%	83 (75)	111
Micello et al. [41]	ER− and PgR−	±	Primary Only	AR27 Novocastra	≥ 1% Nuclear	128 (57)	226
Luo et al. [64]	ER− and PgR−	-	Not Explicitly Reported	Not Reported	≥ 1% Nuclear	38 (28)	137
Niemeier et al. [15]	ER+/ER−	±	Not Explicitly Reported	AR441 DAKO	H score > 10	151(80)	189
Castellano et al. [65]	ER+	±	Primary Only	AR411 DAKO	≥ 1%	609 (71)	859
Hu et al. [28]	ER+/ER−	±	Primary Only	AR411 DAKO	≥ 10% Nuclear	1155 (79)	1467
Loibl et al. [66]	ER+/ER−	±	Primary Only	F39.4.1 BioGenex	≥ 1%	358 (53)	673
Park et al. [27]	ER+/ER−	±	Not Explicitly Reported	AR441 Thermo Scientific	≥ 10% Nuclear	541 (58)	931
Yu et al. [67]	ER+/ER−	±	Not Explicitly Reported	AR441 Lab Vision	≥ 10% Cytoplasmic	237 (72)	327
Gucalp et al. [49]	ER− and PgR−	±	Primary and Metastases	AR411 DAKO	≥ 10% Nuclear	51 (12)	424
Honma et al. [68]	ER+/ER−	±	Not Explicitly Reported	AR27 Novocastra	≥ 10% Nuclear	212 (53)	403
Tokunaga et al. [69]	ER+/ER−	±	Primary Only	AR411 DAKO	≥ 75% Nuclear	155 (62)	250
Thike et al. [60]	ER− and PgR−	-	Not Explicitly Reported	AR27 NCL-AR-318	≥ 1% Nuclear	267 (38)	699
Tsang et al. [70]	ER+/ER−	±	Primary Only	AR441 DAKO	≥ 1% Nuclear	549 (48)	1144
Aleskandarany et al. [71]	ER+/ER−	±	Not Explicitly Reported	Sc-816 Santa Cruz Biotech	H score ≥ 190	613 (54)	1141
Bronte et al. [72]	ER+/ER−	±	Primary and Metastases	SP107 Cell Marque Ventana Medical Systems	≥ 1% and ≥ 10%	136 (83) and 131 (80)	164
Candelaria et al. [73]	ER− and PgR−	-	Not Explicitly Reported	AR441 DAKO	≥ 10%	45 (31)	144
Kensler et al. [13]	ER+	±	Not Explicitly Reported	AR441 DAKO	≥ 1% Nuclear	2475(82)	3021
Xiang et al. [74]	ER+/ER−	±	Primary Only	ZA-0554 ZSGB	≥ 10% Nuclear	201 (67)	298
Zhao et al. [75]	ER− and PgR−	–	Not Explicitly Reported	Abcam ab113273	Not Reported	60 (29)	210

**Table 2:** Completed and ongoing trials assessing the safety and efficacy and targeting androgen receptor in breast cancer

NCT number	Title	BC subtype	AR agent drug class	Therapeutic agent	AR eligibility criteria	Menopausal status	Phase
NCT02463032	Efficacy and safety of GTx024 in patients with ER+/AR+ breast cancer	ER+/AR+	SARM	Enobosarm	Not Defined	Postmenopausal	Phase II
NCT02955394	Preoperative Fulvestrant with or without Enzalutamide in ER+/HER2− breast cancer	ER+/HER2−/AR+	Antiandrogen	Enzalutamide + Fulvestrant	Not Defined	Postmenopausal	Phase II
NCT02910050	Bicalutamide plus aromatase inhibitors in ER+/AR+/HER2− metastatic breast cancer (BETTER)	ER+/HER2−/AR+	Antiandrogen + AI	Bicalutamide + AI	Not Defined	Postmenopausal	Phase II
NCT01616758	Phase II study of GTx024 in women with metastatic breast cancer	ER+ (any AR status)	SARM	Enobosarm	Not Defined	Postmenopausal	Phase II
NCT00755885	Abiraterone acetate in treating postmenopausal women with advanced or metastatic breast cancer	AR+/ER− or ER+ (any AR status)	CYP17-lyase inhibitor	Abiraterone acetate	Not Defined	Postmenopausal	Phase I/II
NCT03207529	Alpelisib and Enzalutamide in treating patients with AR and PTEN+ metastatic breast cancer	HER2−/PTEN+/AR+	Antiandrogen	Enzalutamide + Alpelisib	≥ 1% nuclear staining	All	Phase I*
NCT02580448	CYP17-Lyase and AR inhibitor treatment with Seviteronel trial (CLARITY-01)	TNBC/AR+ or ER+/AR+	CYP17-lyase inhibitor	Seviteronel	Not Defined	Postmenopausal (if ER+)	Phase I/II
NCT02676986	Short-term preoperative treatment with Enzalutamide, alone or in combination with Exemestane in primary breast cancer (ARB)	ER+ and TNBC/AR+	Antiandrogen	Enzalutamide + Exemestane	> 0% nuclear staining	Postmenopausal (if ER+)	Phase II
NCT01990209	Orteronel as monotherapy in patients with metastatic breast cancer (MBC) that expresses AR	ER/PgR+/AR+ or TNBC/AR+	CYP17-lyase inhibitor	Orteronel	> 10% IHC staining	Postmenopausal or Pre with Ovarian Suppression (if ER+)	Phase II
NCT02067741	CR1447 in endocrine responsive-HER2- and TN-AR+ breast cancer	ER/PgR+ (HER2−) or TNBC/AR+	Antiandrogen	CR1447 (4-OH-testosterone)	> 0% IHC staining	Postmenopausal	Phase II
NCT02091960	A study to assess the efficacy and safety of enzalutamide with trastuzumab in patients with HER2+, AR+ metastatic or locally advanced BC	HER2+/AR+	Antiandrogen	Enzalutamide + Trastuzumab	Not Defined	All	Phase II
NCT00468715	Bicalutamide in treating patients with metastatic breast cancer	ER−/PgR−/AR+	Antiandrogen	Bicalutamide	> 10% nuclear staining	All	Phase II
NCT03055312	Bicalutamide in treatment of AR+ metastatic triple-negative breast cancer	TNBC/AR+	Antiandrogen	Bicalutamide	IHC ≥ 10%	All	Phase III
NCT01889238	Safety and efficacy study of enzalutamide in patients with advanced, AR+, triple-negative breast cancer	TNBC/AR+	Antiandrogen	Enzalutamide	Not Defined	All	Phase II
NCT02457910	Taselisib and Enzalutamide in treating patients with AR+ triple-negative metastatic breast cancer	TNBC/AR+	Antiandrogen	Enzalutamide + Taselisib	≥ 10% nuclear staining	All	Phase Ib/II
NCT02605486	Palbociclib in combination with Bicalutamide for the treatment of AR+ metastatic breast cancer	TNBC/AR+	Antiandrogen	Bicalutamide + Palbociclib	≥ 1% nuclear staining	All	Phase I/II
NCT02971761	Pembrolizumab and Enobosarm in treating patients with AR+ metastatic TNBC	TNBC/AR+	SARM	Enobosarm + Pembrolizumab	≥ 50% nuclear staining	All	Phase II
NCT03090165	Ribociclib and Bicalutamide in AR+ TNBC	TNBC/AR+	Antiandrogen	Bicalutamide + Ribociclib	> 0% IHC staining	All	Phase I/II
NCT02353988	AR-inhibitor Bicalutamide in treating patients with TNBC (Arbre)	TNBC/AR+	Antiandrogen	Bicalutamide	> 10% nuclear staining	All	Phase II
NCT02750358	Feasibility study of adjuvant Enzalutamide for the treatment of early-stage AR+ triple-negative breast cancer	TNBC/AR+	Antiandrogen	Enzalutamide	≥ 1% nuclear staining	All	Phase II
NCT03383679	Study on AR- and triple-negative breast cancer (START)	TNBC/AR+	Antiandrogen	Darolutamide + Capecitabine	≥ 10% IHC staining	All	Phase II
NCT02689427	Enzalutamide and paclitaxel before surgery in treating patients with stage I–III AR+ triple-negative breast cancer	TNBC/AR+	Antiandrogen	Enzalutamide + Paclitaxel	≥ 10% nuclear staining	All	Phase II*
NCT05095207	Abemaciclib in combination with Bicalutamide for androgen receptor-positive, HER2-negative metastatic breast cancer	HER2−/AR+	Antiandrogen	Abemaciclib + Bicalutamide	≥ 1% IHC staining	All	Phase Ib/II*
NCT04869943	Efficacy evaluation of Enobosarm monotherapy in treatment of AR+/ER+/HER2− metastatic breast cancer (ARTEST)	ER+/HER2−/AR+	SARM	Enobosarm	≥ 40% nuclear staining	Postmenopausal or Pre with Ovarian Suppression	Phase III*
NCT05065411	Efficacy and safety evaluation of Enobosarm in combo with Abemaciclib in treatment of ER+/HER2− metastatic breast cancer	ER+/HER2−/AR+	SARM	Enobosarm + Abemaciclib	≥ 40% nuclear staining	All	Phase III*
NCT03650894	Nivolumab, Ipilimumab, and Bicalutamide in HER2− breast cancer patients	HER2−/AR+	Antiandrogen	Nivolumab + Ipilimumab + Bicalutamide	Not Defined	All	Phase II*

*Study is Actively Recruiting

## Androgens in breast cancer development

The role of androgens in BC development and progression has been contrasting. While it is well known that androgens can act to inhibit growth in BC, the androgen excess theory proposes that androgens are instrumental in the development of BC in both ER+ and ER− tumours (reviewed in Secreto et al. [17]). Studies have shown that androgens have anti-proliferative properties during puberty and oppose oestrogens, while oestrogen and progesterone promote breast development under physiologic conditions [18, 19]. The equilibrium between oestrogen and androgen allows for the control of mammary epithelial growth. While androgens are growth inhibitory, they are also precursors to oestrogens, so they can also act to stimulate breast cell growth and consequently overstimulate cell proliferation through conversion to oestrogen [17]. Under conditions of excess androgens, the balance between androgen and oestrogen is maintained at a new higher level; however, eventually the stimulatory effects of oestrogens predominate. Through this mechanism, the imbalance of androgen and oestrogen has been shown to lead to the development of ER+ BC. Prospective studies have repeatedly shown a link between high circulating androgens and the development of ER+ BC [20, 21]. Notably, this conversion from androgens to oestrogens is often inhibited through aromatase inhibitors (AIs) in ER+ BC, resulting in increased circulating androgens and decreased levels of oestrogen [22]. Circulating androgens are also associated with a roughly twofold increased BC risk in postmenopausal women. When adjusted for circulating oestrogen to account for the conversion of androgens to oestrogen the association is only partially diminished, confirming an effect of androgens on breast tissue independent of the effect of oestrogen [20, 21, 23].

Androgens and AR expression have also been implicated in the development of ER– BC. AR positivity is shown to be associated with overexpression of HER2 in apocrine tumours, such as tumours of the mammary gland, suggesting an interaction of AR and HER2 signalling pathways in these cells [24, 25]. When apocrine cells are stimulated by androgens, they produce epidermal growth factor (EGF), which results in cell growth and proliferation through stimulation of EGFR and HER2 [17]. Notably, EGFR is often overexpressed in BC, and it is possible that androgen stimulation would lead to the growth of cells under these conditions. Activation of both these pathways provides an opportunity for the development of ER– BC through androgen stimulation. Additionally, ER– BCs that retain AR expression are shown to have gene expression profiles that closely resemble that of ER+ BC.

## AR prognostic value by subtype

### Luminal breast cancer

PAM50 is a commonly used method of classifying intrinsic subtypes of BC using a minimal gene set [26]. Under the PAM50 classification, BC, which is both ER+ and PgR+, is subclassified as luminal A and B. The luminal A are low proliferating and luminal B are divided into HER2+ and HER2–. The majority of luminal BCs express AR by IHC, and this expression is associated with a favourable prognosis [27, 28]. One meta-analysis shows that in early BC, AR is more likely to be co-expressed in ER+ over ER– tumours (74.8% vs. 31.8%, respectively) [29]. AR may act through genomic signalling interference to reduce the proliferation of BC in the presence of oestrogen. One proposed mechanism for growth inhibition in the ER+ BC subtypes is by competitive binding of AR to the ER binding site on DNA [30]. A crosstalk between AR and ER has been proposed, and preclinical data suggest AR can antagonize ER signalling, dependent upon the relative levels of each hormone receptor [31]. Contrastingly, in BCs that do not express ER, AR is able to bind to oestrogen response elements (EREs) on the DNA and stimulate cell proliferation [32]. A large-scale clinical and gene expression meta-analysis from Bozovic-Spasojevic et al. confirmed the findings shown in previous studies where AR positivity conferred improved disease-free survival and overall survival in ER+ BC [33]. Further support for this observation was seen in a retrospective study where patients with tumours expressing both AR and ER had a better prognosis than those with either AR or ER positivity [32]. Additionally, the ratio of the hormone receptors has been suggested as being relevant for prognostic value, with high AR relative to ER proving to be predictive of hormone therapy resistance in early studies [34]. While several studies support the improved prognostic value of AR in ER+ BC, some propose the activation of signalling pathways that would lead to increased proliferation of BC cells. Steroid receptors including AR can have extranuclear functions involved in cell growth and survival. For instance, AR is able to activate cell proliferation through oestradiol stimulation of the formation of a complex between AR, ER and Src. This complex ultimately activates the PI3K/Akt and MAPK pathways [35, 36]. Disruption of the interaction between AR/Src weakens the formation of this complex and can inhibit proliferation [37, 38]. Similarly, EGF signalling was dependent on the formation of this complex, confirming that the AR/ER/Src association plays a role in cell cycle progression [39]. Further studies show that hormone therapy resistance occurs in ER+ models with AR overexpression through EGFR [40].

### HER2-enriched breast cancer

AR is expressed in about 60% of HER2+ BC by IHC [4]. In women with HER2+ BC, findings consistently report a worse prognosis with AR positivity [41]. The mechanism proposed for this unfavourable prognosis is through AR signalling mediated transcriptional induction of ligands involved in Wnt/β catenin and HER2 signalling pathways. AR stimulated WNT7B activation leads to nuclear localization of β catenin and subsequent interaction of β catenin with AR to increase HER3 expression (42). Both Wnt and HER2 signalling pathways have the potential for positive feed-forward activation of AR activity, suggesting an androgen-independent activation of AR in these tumours. This has also been demonstrated in castrate-resistant prostate cancer (CRPC) [43].

The efficacy of treatment of HER2+ AR+ BC with enzalutamide and trastuzumab (HER2 mAb) is currently under investigation in a phase 2 clinical trial (NCT02091960). In contrast to these findings, however, a gene expression analysis has found that overall survival is in fact better for AR mRNA expression in HER2+ BC. These conflicting results could be attributed to there being only modest overlap between transcriptional and IHC profiles for AR [33, 44].

### Triple-negative breast cancer (TNBC)

TNBC is defined as lacking expression of ER, PgR and HER2. This occurs in approximately 15–20% of BCs, but represents a disproportionate rate of mortality, as it is a more aggressive subtype [45]. Quadruple negative breast cancer (QNBC) is broadly TNBC that also does not express AR [46]. Patients with early TNBC suitable for surgery are frequently offered adjuvant chemotherapy, if deemed fit enough. However, these patients continue to have overall poorer survival and higher rates of distant metastasis following treatment [47]. Due to the lack of molecular targets, new therapeutics are needed for this subtype.

TNBC has recently been further categorized based on the gene expression profiles by the Lehmann molecular classification as follows: basal-like (BL1 and BL2), mesenchymal, mesenchymal stem-like, immunomodulatory, and luminal androgen receptor (LAR) [48]. AR positivity is reported between 12 and 50% of TNBC [4, 44, 49]. The existence of an AR-expressing subtype of TNBC along with somatic mutations identified in AR-responsive genes in some sequencing has sparked interest in AR as a target for therapy of this aggressive type [50, 51]. These Lehmann subtypes are shown to respond differently to therapies, with the LAR type being less proliferative and less sensitive to chemotherapy than the basal type [52, 53]. Many studies have shown that AR expression in TNBC is associated with lower histologic grade and lower clinical stage [54–56]. Other groups have shown that a lack of AR expression increases the risk of recurrence and metastasis in TNBC [57, 58]. A retrospective study found AR positivity in TNBC to be associated with improved disease-free survival, while another found LAR TNBC to have higher overall survival [59, 60]. Another recent study stratified patients into distinct TNBC risk groups, finding LAR (AR+, EGFR−) patients to be in a lower-risk group with a better prognosis and likely to benefit most from antiandrogen therapy. Alternatively, AR− and EGFR+ represent the high-risk group that has a worse prognosis and is more likely to benefit from chemotherapy [61]. The prognostic value of AR in TNBC is still uncertain, with some reports suggesting a positive prognosis with AR expression and others reporting no effect. This is likely variable based on the molecular profile of the cancer as well as the clinical context (Reviewed in Bozovic-Spasojevic et al. meta-analysis [33]). Understanding the prognostic value of AR under these variable molecular and clinical contexts will be instrumental in using AR-targeting therapies for the treatment of BC.

### Apocrine breast cancer

Apocrine BCs are not classified as a distinct subtype within the PAM50 classification and they make up only around 1% of all BCs [76]. Apocrine BCs are ER/PgR– and generally, but not always, HER2−. Because of this and their uncommonness, apocrine BCs are generally grouped with TNBCs despite having distinct morphology. These tumours are characterized by having apocrine cells with oeosinophilic and granular cytoplasm and a low nuclear–cytoplasmic ratio. Additionally, activation of the AR signalling pathway is a prominent feature in apocrine BC [77]. Apocrine BCs are ER/PgR– and therefore may be either HER2-enriched or, more frequently triple-negative. Importantly not all AR+ but ER/PgR– BCs are apocrine.

Triple-negative apocrine BC frequently carry actionable genomic alterations including alterations in *PIK3CA* and *PTEN* [78]. Approximately 80% of invasive apocrine tumours occur in postmenopausal women [79]. Due to its relatively low prevalence, it remains unclear whether the clinical features and outcomes of apocrine BC differ significantly from non-apocrine BC (either AR+ or AR–). Recently, an AR+ apocrine cell line model was shown to have an AR-dependent proliferative response to androgens and expression of genes that are normally expressed in ER+ luminal tumours [44]. Further work showed that in this model AR binds and regulates ER cis-regulatory elements through a FoxA1-dependent mechanism leading to the gene expression profile overlap with ER+ BC [80]. Apocrine BC’s strong association with AR highlights their importance as a model to understand AR signalling in BC, and these tumours should be enriched for clinical trials investigating AR-targeted agents.

## Androgen-targeting therapy

**Fig. 1:**
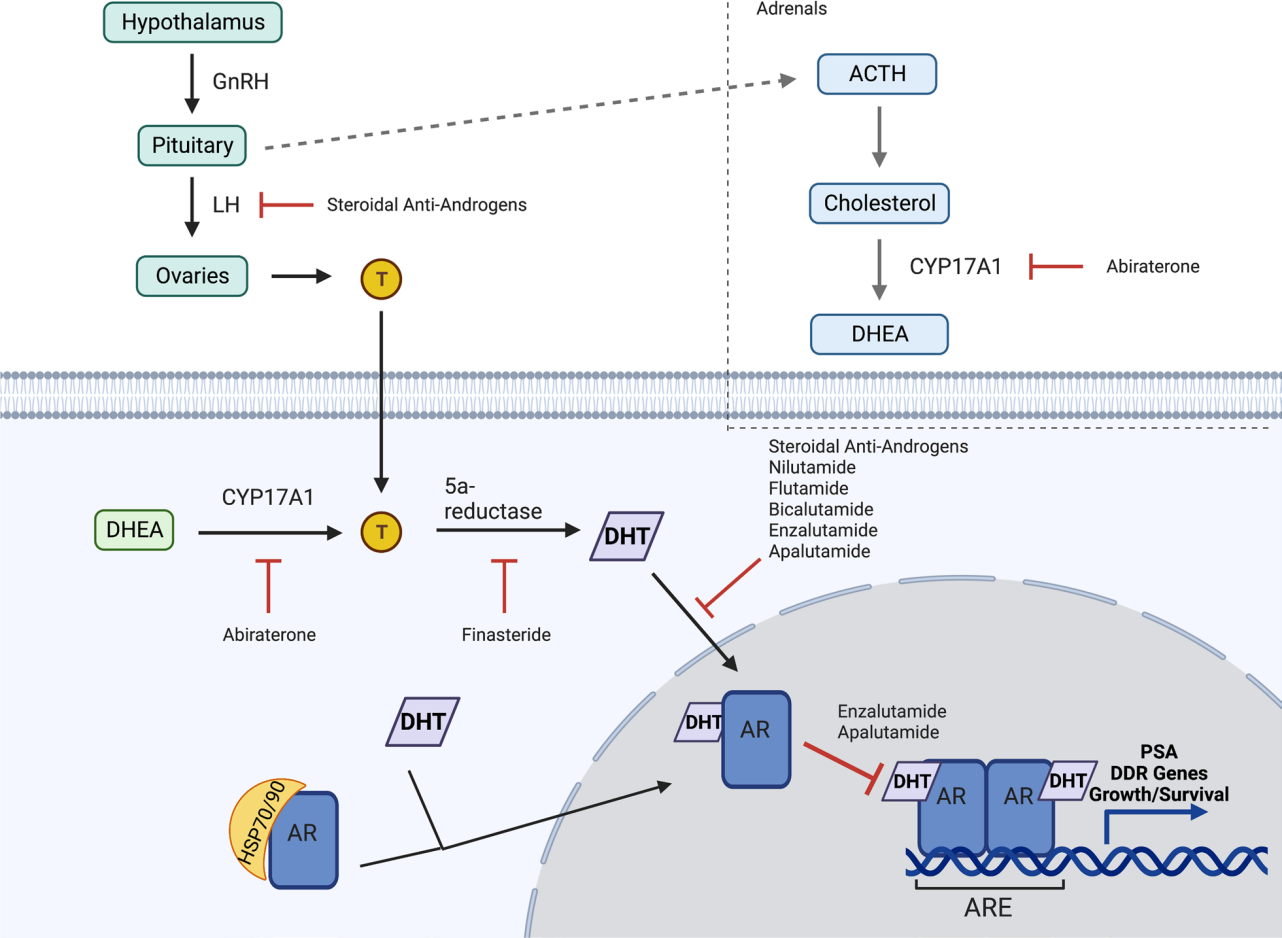
AR signalling pathways and therapeutic targets of AR. Androgens such as T are produced from cholesterol. CYP17A1 is the enzyme responsible for converting precursors to DHEA, and T is converted to DHT through 5a-reductase. DHT activates AR, resulting in its release from HSP70/90. AR then dimerizes and translocates to the nucleus where it can bind to AREs and modulate the transcription of target genes. Abiraterone irreversibly inhibits CYP17A1 activity. Bicalutamide and enzalutamide are AR antagonists that block the binding of DHT to AR. Enzalutamide also inhibits the nuclear translocation of AR. Abbreviations: GnRH—gonadotropin-releasing hormone, LH—luteinizing hormone, T—testosterone, ACTH—adrenocorticotropic hormone, DHEA—dehydroepiandrosterone, ARE—androgen response element, and PSA—prostate-specific antigen

Targeting androgens in PCa has been a goal of treatment since the 1940s, with new classes of antiandrogens developed as knowledge of androgen biosynthesis and signalling has increased (Fig. 1). Nonsteroidal antiandrogens were developed to target AR without the nonspecific effects of their steroidal predecessors. While these drugs are safer, they have a disadvantage of lower affinity for AR, leaving about 5–10% of DHT uninhibited and able to bind and activate AR. Newer generations of antiandrogens were developed to address this issue. These next-generation agents include abiraterone, an inhibitor of cytochrome P17 (CYP17), and enzalutamide. CYP17 is required in the androgen biosynthesis pathway, and its inhibition leads to decreased levels of DHT. The history and development of antiandrogen therapies are described more extensively in other reviews [81].

Evidence of a role for androgens and AR in BC development and progression has led to considerable interest in AR as a potential therapeutic target. Antiandrogens such as bicalutamide and enzalutamide have shown early success in preclinical and clinical trials of antitumour response, and further trials have enrolled both ER– and ER+ anti-oestrogen-resistant patients [24, 82, 83]. The goal of using ADT as a therapy in these patients is to block the activation of AR and associated pathways such as ErbB that are involved in BC progression. However, anti-AR strategies in BC have yet to be established due to a lack of consistent positive trial data and, as detailed below, this can partly be explained by the biological complexity of AR signalling in BC.

In contrast to strongly AR+ TNBC, low-level AR expression has been associated with more aggressive forms of TNBC, suggesting that in at least some subtypes of BC, ADT may have a tumour-promoting effect [84]. Cochrane et al. presented clinical data that a high nuclear ratio of AR relative to ER in patients treated with tamoxifen (an anti-oestrogen therapy) predicted failure in therapy. They then went on to show that enzalutamide treatment decreased growth in both ER+ and ER−/AR+ tumours, suggesting a role for antiandrogens in hormone resistant cancers [85]. They were the first to show that androgen-targeted therapies could have clinical benefit in ER– BC, either alone or in combination with tamoxifen and/or AIs. They also suggest a role for targeting AR in recurrent ER+ BC, where selective targeting of the ER pathway could lead to the tumour cells switching to androgen dependence.

Clinically, however, data do not support AR as a biomarker for selecting anti-oestrogen therapy. Results from NCT00004205 show that in postmenopausal ER+ BC patients, AR expression did not predict treatment efficacy of anti-oestrogen therapy monotherapy. Alternatively, AR expression has been shown to be useful in predicting the efficacy of antiandrogen therapy, including AR positivity in TNBC predicting response to enzalutamide [86, 87]. While much attention has been focused on drugs that block androgens, research is also looking into the efficacy of 17α hydroxylase/17–20 lyase (CYP17) inhibitors (i.e. abiraterone) that block androgen, oestrogen and glucocorticoid synthesis. Early results from these trials show mixed responses and the studies are ongoing. These studies are covered in more detail by other reviews [24, 82, 83].

There are currently multiple clinical trials evaluating the use of AR antagonists in BC. In one study of AR+ TNBC, patients were treated with bicalutamide 150 mg daily. The results showed clinical benefit (defined as a complete or partial response or stable disease) of 19%, with a median progression-free survival of 12 weeks [49]. This study was the first proof that AR antagonist treatment is beneficial in AR+ TNBC. Additional phase II trials are ongoing testing bicalutamide and enzalutamide in AR+ TNBC, showing a clinical benefit of 20% for bicalutamide (NCT00468715) and 28–33% for enzalutamide [86]. Further studies are evaluating enzalutamide in combination with other therapeutics such as trastuzumab in AR+ /HER2+ BC (NCT02091960), and paclitaxel in early-stage AR+ TNBC (NCT02689427). Many of these trials show promise of alternate therapeutic options for TNBC beyond cytotoxic chemotherapy alone.

While most AR-targeted therapies are aimed at inhibiting signalling, there is evidence of AR activation producing growth repression. This has been well demonstrated in some PCas, especially those that have adapted to low androgen environment growth. In the case of PCas, there is typically a biphasic growth where either ADT or supraphysiologic androgens produce growth suppression [88, 89]. This has been replicated in BC exposed to high concentrations of oestrogen [90, 91]. Several mechanisms have been proposed for the observation of growth repression at high concentrations of androgens including cMyc activation and activation of negative regulators of the cell cycle [92]. High doses of androgens have been shown to induce DNA damage. This is consistent with previous reports that AR activation results in transient double-strand DNA breaks to release DNA topologies that inhibit the function of RNA polymerase during transcription [93]. Because of its ability to promote DNA damage, AR is also a promising target for combined therapeutics with radiation (discussed later). Due to the ability of AR to act as either a positive or negative regulator of cell growth and proliferation depending on the molecular features of the BC and the presence of oestrogens, both AR agonists and antagonists are being actively tested as potential therapies. While synthetic androgens have proven to have undesired side effects, selective AR modulators (SARMs) have promising preclinical effects on reducing tumour burden in ER+ BC and have fewer side effects [94]. Recently it was shown in a patient-derived xenograft (PDX) model of BC that treatment with a SARM, but not an AR antagonist, inhibited cell proliferation. This was further shown to occur through a reprogramming of the ER cistrome to an AR cistrome, likely through a FOXA1-dependent mechanism [95]. This was further corroborated by Hickey et al. findings that AR agonist treatment could be combined with standard of care in ER+ BC to enhance antitumour response. They showed that activation of AR in this context alters the genomic distribution of ER as well as other co-activators leading to decreased expression of ER-regulated cell cycle genes and upregulation of AR-regulated tumour suppressor genes [96]. Evaluation of SARMs in ER+ and AR+ BC is currently under further investigation in a phase II trial (NCT02463032).

## AR and DNA damage repair

Steroid hormone receptors such as ER in BC are known to be involved in regulating the DNA damage response (DDR), and suppression of ER signalling with tamoxifen has been shown to augment RT response [97–99]. Goodwin et al. presented a mechanism by which this association between steroid hormones and DNA repair extends to AR activity in a PCa model [100]. In men with localized PCa, the standard of care is a combination of ADT with radiation, as the combination has been shown to be more effective than either treatment on its own [101–103]. AR activity allows for DNA DSB resolution, independent of cell cycling effects, by regulating some of the accessory factors necessary for DDR. AR acts as a transcriptional regulator of DDR factors, including DNA-PKcs, a kinase important in non-homologous end joining (NHEJ), to increase DNA repair and cell survival following DNA damage induction. Furthermore, DNA-PKcs then create a positive feedback circuit wherein AR is further activated to increase its transcription-activating potential [100]. Polkinghorn et al. confirmed this role of AR as a transcriptional regulator of DNA repair genes, and they showed that treatment with ADT decreases the expression of these genes [104]. Additional studies have also implicated a role for an AR-PARP1 signalling axis in breast cancer and have shown that combined inhibition of AR and PARP leads to enhanced antitumour activity by modulating the DDR [105, 106]. The implications for a role of AR in DDR make it an interesting target for combination therapeutics with RT, as AR inhibition should potentiate radiation-induced damage in a tumour-selective manner. It should also be noted, however, that while AR signalling allows for damage repair, supraphysiological androgens are associated with the induction of DSBs [107]. For this reason, supraphysiological androgens are also under evaluation as a method of augmenting radiation response. This is actively being studied in PCa, and while it may be useful in BC, its use may be limited by side effects seen in previous studies with androgen agonists in BC.

## Radiation therapy and AR

RT along with pharmacotherapy and surgery continues to play an important role in the treatment of BC. RT following breast-conserving surgery halves the rate at which the disease recurs and reduces the BC death rate by approximately a sixth [108]. While this multimodal treatment approach has proven to be effective for many women, some will still go on to develop recurrent disease, including many women diagnosed with TNBC. Speers et al. showed that patients with TNBC who have AR expression above the median were more likely to have locoregional recurrence following RT; however, there was no difference in locoregional recurrence in the TNBC patients who were not treated with RT. Importantly, this was shown in retrospective data sets and therefore it cannot be assumed that the clinicopathological status of the patients in the RT-treated and non-RT-treated groups are comparable [109]. A retrospective study of BC found that following RT, relapsed tumours were more likely to express AR than non-relapsed tumours [110]. Additionally, Yard et al. found that within a subgroup of 28 BC cell lines from an RT resistance screen AR mRNA levels were correlated to relative RT resistance. They went on to show that AR+ BC cells that were treated with DHT before RT had less DNA damage than cells treated with enzalutamide before RT. They attributed this to increased activation of DNA-PKcs [111].

As previously discussed, TNBC is generally associated with poor survival despite RT and chemotherapy and these patients also suffer increased rates of locoregional recurrence [112–114]. Consequently, strategies that can improve responses to RT in these patients would be of significant clinical value. A subgroup of TNBC is known to express AR, and this group has been shown to be susceptible to targeting of the AR [48]. Clinical trials are currently underway to assess this blockade in patients with metastatic BC expressing AR (NCT00468715, NCT03055312, NCT00755885, NCT01889238, NCT02580448—clinicaltrials.gov). Further studies have demonstrated that targeting AR may be useful not only as a monotherapy, but as a means of radiosensitizing TNBC [100, 104, 109, 115]. It is suggested that inhibiting AR with enzalutamide in AR+ TNBC (as well as in PCa) can decrease the potential for DNA repair, leading to amplified damage from RT and ultimately cell death. This has been hypothesized to occur, in part, due to an abrogation of the expression of DNA-PKcs [100, 104, 109]. This radiosensitization has also been observed using seviteronel in AR+ TNBC, where radiosensitization was induced due to delayed DSB repair [116]. The potential for AR-targeting- therapies as a means of radiosensitizing TNBC is under active investigation and remains a promising therapeutic strategy for this subtype.

## Conclusions

While AR continues to show promise as a biomarker and a therapeutic target in BC, its role in the modern-day management of patients remains uncertain. The previous sections illustrated that the prognostic value of AR may differ based on the clinical BC subtype (primarily based on PAM50 criteria). However, the precise association of AR positivity with prognosis remains unclear. One possible explanation for this may be that AR positivity, for instance as characterized by AR gene expression, does not accurately predict AR activity. Given the differences in biology and prognosis across BC subtypes, in order to accurately test the association between AR expression and activity, the method by which the BC subtypes are classified is of critical importance. To date, BC subtypes have primarily been characterized using PAM50 criteria which are molecularly stratified based on gene expression profiling and relate to oestrogen and progesterone hormone receptor status. While this classification system is useful, it has its limitations, namely substantial variation within groups and lack of representation of rarer subtypes [117, 118]. Based on the understanding that much of the gene expression landscape is driven by copy number alterations (CNAs), a new classification system for BC was developed [119].

The Molecular Taxonomy of Breast Cancer International Consortium (METABRIC, www.cbioportal.org) used a combination of gene expression and CNAs to stratify BC into 10 integrative clusters (IntClust/s). These IntClusts cover the subtypes defined using other approaches; however, they also include novel tumour subtypes [120, 121]. The IntClusts represent distinct clinical characteristics and response to therapeutics, and one may hypothesize that assessing AR positivity across clusters may help to resolve some of the conflicting prognostic significance seen when stratifying by PAM50 criteria. While more detailed in silico analysis, followed by validation in preclinical and clinical data sets is required to fully evaluate AR in BC IntClusts, we propose that the IntClust classification system may provide additional value in understanding the biological and clinical significance of AR expression in BC. This deeper understanding will support the characterization of BC subtypes in which AR status is more prognostic of outcome and help identify subtypes where therapeutically targeting AR may be most effective. Recent work has given more clarity on the clinical role of AR in ER+ BC; however, uncertainties remain in the other disease subtypes. It can be argued that understanding AR as it relates to the genomic landscape of BC, as classified by integrative clusters, would be a more nuanced way of stratifying patients for AR therapy. Alternatively, biomarkers that capture active AR signalling driving tumorigenesis/tumour maintenance may be necessary to faithfully identify patients most likely to benefit from AR-directed therapy. However, identifying and validating these biomarkers is unlikely to be trivial and may require interrogation of both transcriptional regulation and gene expression through methods such as chromatin immunoprecipitation (ChIP) and RNA sequencing. Targeting AR has shown promising results in multiple clinical trials, along with androgen-targeting therapies in combination with other therapies, such as tamoxifen in ER+ BC or RT in TNBC. Harnessing the full potential of targeting AR will require a more refined understanding of the role of AR in each subtype of BC.

## Data Availability

Not applicable.

## Abbreviations

ADT: Androgen deprivation therapy
AI: Aromatase inhibitor
AR: Androgen receptor
ARE: Androgen response element
BC: Breast cancer
ChIP: Chromatin immunoprecipitation
CNA: Copy number alteration
CRPC: Castration-resistant prostate cancer
CYP17: Cytochrome P17
DDR: DNA damage repair
DHEA: Dehydroepiandrosterone
DHT: 5A-dihydrotestosterone
DSB: Double-strand break
EGF: Epidermal growth factor
ER: Oestrogen receptor alpha
HER2: Human epidermal growth factor receptor 2
IHC: Immunohistochemistry
IntClust: Integrative cluster
NHEJ: Non-homologous end joining
PARP: Poly(ADP-ribose) polymerase
PCa: Prostate cancer
PgR: Progesterone receptor
QNBC: Quadruple negative breast cancer
RT: Radiation therapy
SARM: Selective AR modulator
T: Testosterone
TNBC: Triple-negative breast cancer
